# Modification of light absorption in thin CuInS_2_ films by sprayed Au nanoparticles

**DOI:** 10.1186/1556-276X-9-494

**Published:** 2014-09-14

**Authors:** Atanas Katerski, Erki Kärber, Ilona Oja Acik, Leonid Dolgov, Arvo Mere, Ilmo Sildos, Valdek Mikli, Malle Krunks

**Affiliations:** Laboratory of Thin Film Chemical Technologies, Department of Materials Science, Tallinn University of Technology, Tallinn, EE-19086 Estonia; Institute of Physics, University of Tartu, Tartu, EE-50411 Estonia; Centre for Materials Research, Tallinn University of Technology, Tallinn, EE-19086 Estonia

**Keywords:** CuInS_2_ thin films, Chemical spray pyrolysis, Au nanoparticles, Light absorption

## Abstract

The chemical spray pyrolysis method was used to deposit CuInS_2_ (CIS) thin films and Au nanoparticles (NPs) in two configurations: glass/Au-NP layer covered with CuInS_2_ film (Au-NP/CIS) and glass/CuInS_2_ films covered with Au-NP layer (CIS/Au-NP). According to X-ray diffraction (XRD), the spray of 2 mM HAuCl_4_ aqueous solution with a volume of 2.5 to 15 ml onto a glass substrate at 340°C results in metallic Au nanoparticles with a similar mean crystallite size in the range of 30 - 38 nm. The mean crystallite sizes remain in the range of 15 - 20 nm when grown onto a CIS film. The prepared films show plasmonic light absorption with increasing intensity in the spectral range of 500- 800 nm when increasing the volume of HAuCl_4_ solution sprayed. When compared to bare CIS on glass, the absorptance was increased *ca*. 4.5 times in the case of glass/Au-NP/CIS and *ca.* 3 times in the case of glass/CIS/Au-NP configuration. The glass/Au-NP/CIS configuration had an advantage since Au-NP could be embedded without chemically damaging the CIS.

## Background

Photovoltaics (PV) is a clean and sustainable resource for producing electricity from sunlight. The future success of PV electricity requires significant advances in materials research and advances in the structural design of solar cells to increase conversion efficiency of the cell and reduce manufacturing costs [1]. In view of manufacturing costs, PV devices such as dye-sensitized solar cells (DSSC), solar cells that use organic absorber, and extremely thin inorganic absorber (eta) solar cells are promising as prepared using low-cost non-vacuum technologies. However, all of these solar cells are based on ultrathin absorber layers, which in turn, lead to poor absorption of sunlight and low conversion efficiency when compared with conventional thin film solar cells. The plasmonic solar cell concept has been proposed in which metallic nanoparticles are embedded in the solar cell to increase light absorption ability and by that to enhance the conversion efficiency in various types of solar cells [2, 3]. Increased attenuation of light can be achieved throughout the visible up to infrared light as long as the embedded nanoparticles are with suitable sizes [4, 5]. Therefore, the addition of metallic nanoparticles to a PV absorber offers a way of reducing the physical thickness of the absorber layers while keeping their optical thickness similar [6]. Plasmon-enhanced light absorption, increased photocurrent, and increased efficiency have been demonstrated in solar cells that use ultrathin light absorbing material, such as DSSC [7, 8] and organic solar cells [1]. The metal (Au, Ag) nanoparticles have been made by methods such as heat treatment and evaporation technique [9], colloidal dispersion [10], spin-coating [11, 12], electrodeposition [13], and spray pyrolysis [14–17].

Eta solar cells use an absorber with a thickness of few tens up to a few hundred nanometers. Eta cells with chemically sprayed CuInS_2_ absorber layer are based either on TiO_2_ nanoparticles [18] or ZnO nanorod layer [19, 20] and show light-to-electricity conversion efficiencies of *ca.* 7% [18] and *ca.* 4% [20, 21], respectively. To obtain Au nanoparticles by chemical spray pyrolysis (CSP), a solution of an Au salt such as HAuCl_4_ · nH_2_O is deposited onto a preheated substrate*.* Thermoanalytical study has shown that the decomposition of HAuCl_4_ · nH_2_O into pure gold and gaseous products is completed at 320°C in air [22]. Recently, it has been shown that SnO_2_[16], ZnO [17], ZrO_2_[14], and TiO_2_[23] thin films with plasmonic nanoparticles can be prepared by CSP at a substrate temperature of 400°C or higher. The formation of Au nanoparticles on a glass substrate has been studied by varying the concentration of HAuCl_4_ in an aqueous solution using the ultrasonic spray method and a substrate temperature of 300°C [15].

In this study, we prepare Au nanoparticles by spray of HAuCl_4_ solution onto a glass and onto the surface of preliminarily grown thin CuInS_2_ film. We study the possibility to observe the surface plasmon resonance effect in two configurations of CuInS_2_ and Au nanoparticle composites in order to increase light absorption in CuInS_2_. To preserve the simplicity of preparation, we use the CSP method for deposition of both components in the composite.

## Methods

A thin CuInS_2_ (CIS) film was grown by CSP of an aqueous solution containing CuCl_2_, InCl_3_, and SC(NH_2_)_2_ as Cu, In and S sources, respectively, at a molar ratio of Cu:In:S = 1:1:3 ([Cu^2+^]/[In^3+^] = 1.0, [Cu^2+^] = 2 mM). The surface temperature of the substrate was kept at 310°C. Other deposition parameters such as spray solution volume of 5 ml and feeding rate of 1 ml/min were kept constant for all samples. These deposition conditions resulted in CuInS_2_ thin films with thicknesses of *ca.* 150 nm.

Gold(III) chloride trihydrate (HAuCl_4_ · 3H_2_O, 99.9%, Sigma-Aldrich, St. Louis, MO, USA) was used as the precursor for the preparation of gold nanoparticles (Au-NPs) by CSP. HAuCl_4_ · 3H_2_O was dissolved in deionized water to obtain solutions with a concentration of 2 mM. The solution was pneumatically sprayed in air onto a bare glass sheet or onto a glass sheet with a thin CIS layer. The substrate surface temperature was 340°C, the solution volume was varied from 2.5 to 15 ml, and the solution feeding rate was 1 ml/min. The Au nanoparticle deposition temperature of 340°C was chosen as the most suitable to obtain films that do not contain unwanted residues from the Au precursor.

**Figure 1:**
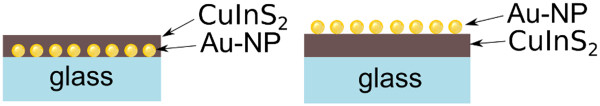
**Sketches of Au nanoparticle (NP) and CuInS**_**2**_ **(CIS) nanocomposite film configurations. (a)** Glass/Au-NP/CIS and **(b)** glass/CIS/Au-NP.

The composite samples that consisted of Au-NPs and the CIS were prepared in two configurations (Figure 1). For the bottom configuration (Figure 1a), the Au-NP layer was deposited firstly onto a soda-lime glass followed by the deposition of the CIS thin film. For the top configuration (Figure 1b), the CIS thin film was deposited firstly followed by the deposition of Au nanoparticles.

The Au-NP layer, the CuInS_2_ film, the Au-NP layer covered with CuInS_2_ film (Au-NP/CIS), and the CuInS_2_ films covered with Au-NP layer (CIS/Au-NP), all on glass substrates, were characterized using optical transmittance and reflectance spectra, scanning electron microscopy (SEM), and X-ray diffraction (XRD) methods.

The total transmittance and the total reflectance spectra of the samples were measured in the wavelength range of 300-1,500 nm on a Jasco V-670 UV-vis-NIR spectrophotometer (Jasco Corporation, Ishikawa-cho, Hachioji-shi, Tokyo, Japan) that was equipped with an integrating sphere to collect the diffused light. The sizes of Au nanoparticles were evaluated from the film surface images and the film thicknesses from the cross-sectional images by SEM. SEM study was performed using EVO MA 15 Zeiss apparatus (Carl Zeiss, Inc., Oberkochen, Germany) at an operating voltage of 10 kV. XRD patterns were recorded in the 2*θ* range of 20° - 70° on a Rigaku Ultima IV diffractometer (Cu Kα radiation, *λ* = 1.5406 Å, 40 kV at 40 mA; Rigaku, Shibuya-ku, Tokyo, Japan) equipped with a silicon strip detector D/teX Ultra. The mean crystallite size of Au particles was calculated from the full width at half maximum (FWHM) of the (111) peak of the metallic Au (PDF 00-004-0784) [24] using the Scherrer formula and the Scherrer constant of 0.94.

## Results and discussion

### Structural properties

**Figure 2:**
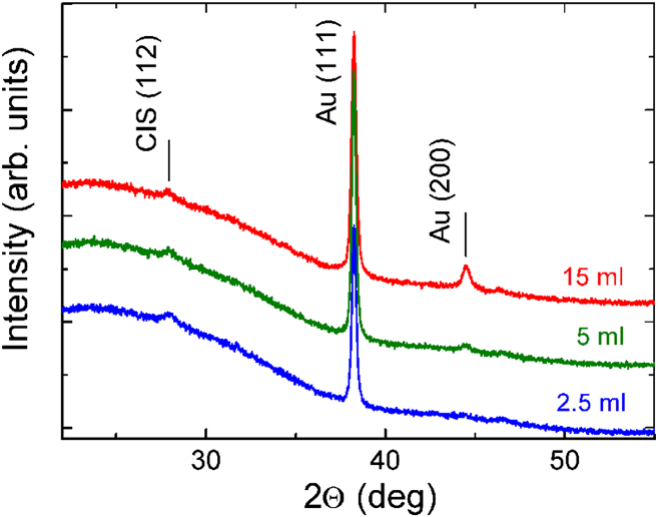
**XRD patterns of Au-NP/CIS nanocomposite films.** Au nanoparticles are prepared by spray of 2.5, 5, and 15 ml of 2 mM HAuCl_4_ aqueous solution at 340°C. CIS was grown by spray onto the Au-NP layer at 310°C.

**Figure 3:**
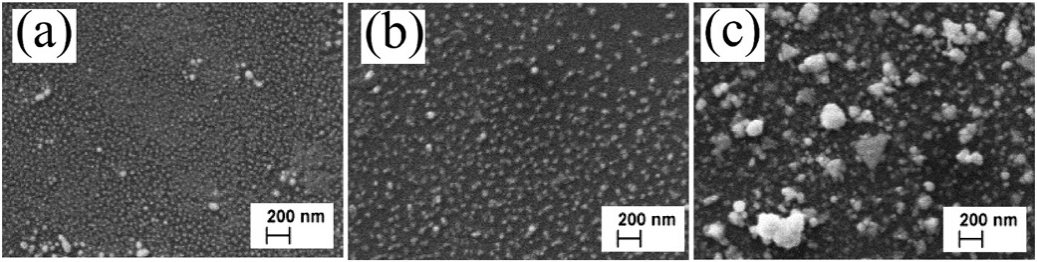
**SEM images of Au nanoparticles on glass.** Au nanoparticles were obtained by spray of 2 mM HAuCl_4_ aqueous solution onto a substrate at 340°C using solution amounts of **(a)** 2.5 ml, **(b)** 5 ml, and **(c)** 15 ml.

Figure 2 presents the XRD patterns of Au-NP/CIS films (see Figure 1a). The diffraction peaks at 2*θ* of 38.2° and 44.4° correspond to the reflections from the (111) and (200) planes of metallic Au with cubic structure (PDF 00-004-0784) [24]. Thus, the spray of HAuCl_4_ aqueous solution at 340°C results in metallic Au phase which according to the SEM study is a layer of nanoparticles (see Figure 3). The formation of metallic gold particles is in good correlation with that reported in literature [15]. The intensities of XRD peaks characteristic of Au are increasing with the solution amount sprayed. The mean crystallite size of Au particles varies slightly, from 36 to 30 nm with no correlation when spraying 2.5 or 15 ml of Au solution (see Table 1). The height of the (112) diffraction peak of CuInS_2_ is similar in all Au-NP/CIS films which could be expected since a similar amount (5 ml) of CIS precursor solution was sprayed.

**Table 1:** **Au nanoparticle size and Au mean crystallite size in nanoparticles**

Configuration	HAuCl _4_ solution volume (ml)	Nanoparticle size (nm)	Crystallite size (nm)
Glass/Au-NP	2.5	30 to 50	36
	5	50 to 80	38
	15	25 to 200	30
Glass/CIS/Au-NP	2.5	20 to 60	15
	10	20 to 60	19
	15	20 to 200	20

The size of Au nanoparticles on glass (glass/Au-NP) and on CIS (glass/CIS/Au-NP) as evaluated from SEM images, and the mean crystallite size of Au as calculated from the FWHM of the (111) diffraction peak of Au according to the Scherrer formula. Au nanoparticles were deposited by spray pyrolysis of 2 mM HAuCl_4_ solution at 340°C.

**Figure 4:**
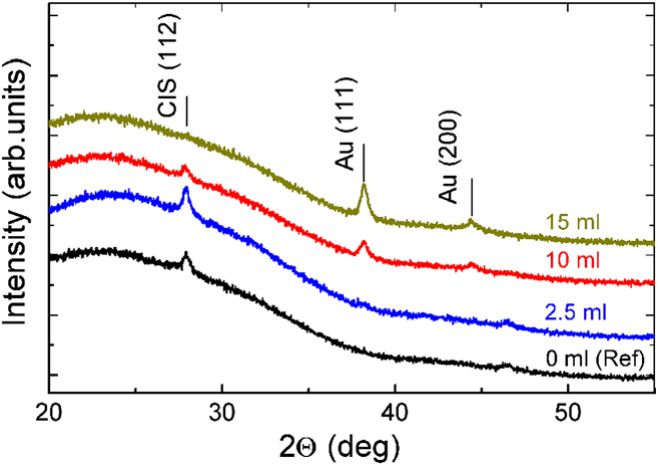
**XRD patterns of CIS film as a reference and CIS/Au-NP nanocomposite films.** Au nanoparticles are prepared by spray of 2.5, 10, and 15 ml of 2 mM HAuCl_4_ aqueous solution at 340°C onto CIS film previously grown at 310°C.



The XRD patterns of the CIS film and of the CIS/Au-NP film (see Figure 1b) are presented in Figure 4. Both the CIS film and CIS/Au-NP film (Au-NPs from 2.5 ml of Au solution) reveal diffraction peaks at 2*θ* of 27.9° and 46.5°. The peaks were assigned to the reflections from (112) and (220) planes of CuInS_2_ (PDF 00-027-0159) [24]. An increase of the Au precursor solution volume from 2.5 to 15 ml leads to a higher intensity of the diffraction peaks characteristic of metallic Au (at 2*θ* of 38.2° and 44.4°) while the mean Au-NP crystallite size remains in between 15 and 20 nm (Table 1). Thus, Au-NP crystallite sizes (on CIS) are smaller compared to those on bare glass substrates. While increasing the spray volume up to 15 ml, the intensities of the diffraction peaks characteristic of CuInS_2_ are vanishing. At 15 ml of Au precursor solution sprayed, the (112) diffraction peak of CuInS_2_ has almost disappeared, indicating the absence of crystalline CIS. Possibly, the thermal decomposition products of HAuCl_4_ such as Cl_2_ and HCl [15, 22] react destructively with CuInS_2_. Presumably, the disappearance of the CIS phase is caused by the following chemical reaction:

However, further studies are needed to prove this reaction.

### Surface morphology

Figure 3 shows the SEM images of Au nanoparticles grown on glass substrates using various amounts of Au precursor solution. As seen, the spray of 2.5 ml HAuCl_4_ solution resulted in Au-NPs with sizes of 30 to 50 nm (Figure 3a, Table 1). By increasing the HAuCl_4_ solution amount to 5 ml, Au-NPs with sizes of 50 to 80 nm are grown (Figure 3b, Table 1). Further increase in the Au precursor solution volume to 15 ml resulted in Au-NPs with the size of 25 to 80 nm and the formation of Au-NP agglomerates with the size of *ca.* 200 nm (Figure 3c, Table 1). According to the literature, the size of Au nanoparticles on the glass substrate deposited by ultrasonic spray has been found to increase from 17 to 47 nm when increasing the concentration of the HAuCl_4_ solution from 1 to 30 mM [15].

**Figure 5:**
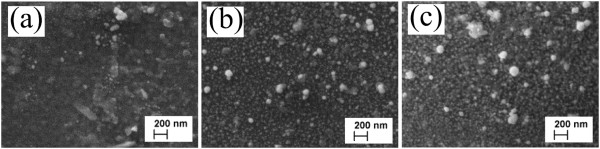
**SEM images of CIS thin films covered with Au nanoparticles.** Au nanoparticles were obtained by spray of 2 mM HAuCl_4_ aqueous solution at 340°C onto CIS films using solution amounts of **(a)** 2.5 ml, **(b)** 10 ml, and **(c)** 15 ml.

The SEM images of Au nanoparticles deposited by spray on top of the previously grown CIS film (CIS/Au-NP) are presented in Figure 5. The spray of 2.5 ml of 2 mM HAuCl_4_ solution resulted in Au nanoparticles with a size of 20 to 60 nm on the CIS film (Figure 5a, Table 1). Increasing the amount of HAuCl_4_ solution to 10 ml resulted in a more dense coverage of the CIS films with Au particles (Figure 5b), while the mean size of the particles remains unchanged (20 to 60 nm). Further increasing the solution amount to 15 ml (Figure 5c) resulted in Au nanoparticles with the size of *ca.* 20 nm as well as in agglomerates with the size of *ca.* 200 nm (Table 1).

### Optical properties

**Figure 6:**
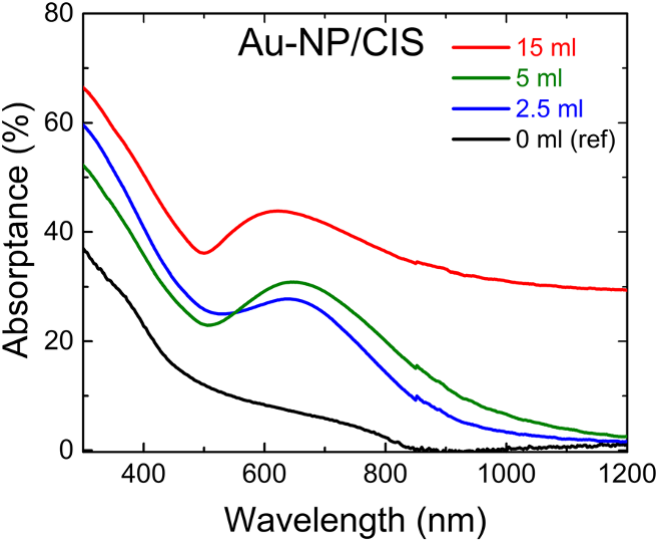
**Optical absorptance spectra of CIS film on glass (ref) and of Au-NP/CIS nanocomposite films on glass.** Au nanoparticles were formed by spraying 2 mM HAuCl_4_ aqueous solution with a volume of 2.5, 5, and 15 ml onto a glass substrate at a temperature of 340°C. CIS was grown by spray onto glass (reference) or Au-NP layer at 310°C.

**Figure 7:**
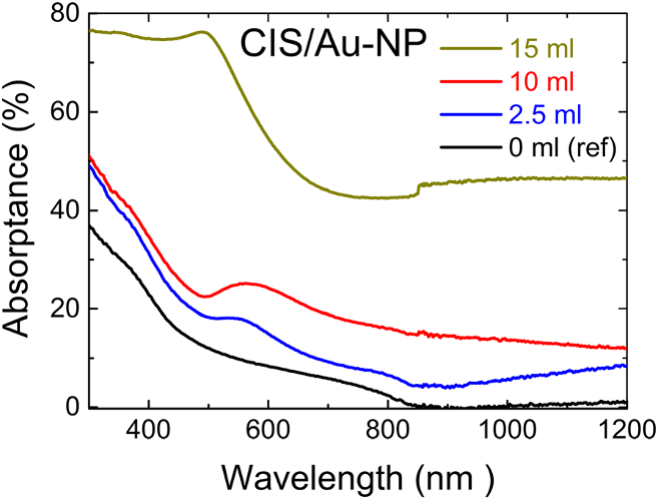
**Optical absorptance spectra of CIS film on glass (ref) and of CIS/Au-NP nanocomposite films on glass.** Au nanoparticles were formed by spraying 2 mM HAuCl_4_ aqueous solution with a volume of 2.5, 10, and 15 ml at 340°C onto the CIS film grown at 310°C.

The optical absorptance spectra of the Au-NP/CIS and CIS/Au-NP layers are presented in Figures 6 and 7, respectively. An additional band appears in the absorptance spectrum of Au-NP/CIS samples between 500 and 800 nm (Figure 6) when compared to the absorptance of the CIS reference film without any embedded Au-NP (denoted as 0 ml, reference). The additional absorption band is attributed to the surface plasmon effect [6, 25]. Here, the Au nanoparticles act as a grid that preferentially diffuses light into the CIS. The use of 2.5- or 5-ml volume of the Au precursor solution results in absorptance up to 30% at 650 nm, while the use of 15-ml solution show further increase up to 45% at *ca.* 600 nm. In the latter, Au nanoparticles are up to four times larger in size (Table 1, Glass/Au-NP) which result in a more dense surface coverage when compared to Au coverage in layers deposited at lower volumes. Since the absorptance *A* was calculated as *A* = 100% - (*R* + *T*), where the total reflectance *R* and the total transmittance *T* include the diffused light, the effect of the surface morphology on the absorptance has been minimized. Thus, a more dense arrangement due to the larger gold particles is likely to cause the strong absorption gain in the case of the 15-ml volume used (Figures 6).

When compared to the absorptance of the structure presented in Figure 1a (Au-NP/CIS), the use of the structure in Figure 1b (CIS/Au-NP) leads to a significantly lower gain of optical absorptance, evident for smaller volumes (2.5 ml) of the Au precursor solution sprayed (Figure 7). Since in the CIS/Au-NP configuration, the gold particles are only partially in contact with the CIS, the absorptance is less enhanced. When using a solution volume of 15 ml, a much higher absorptance above 70% is present at around 500 nm. However, at 15 ml, no crystalline CIS was detected (Figure 4); thus, the absorptance is not of the CIS/Au-NP sample and is difficult to interpret.

We have shown that the use of Au-NP/CIS configuration (Figure 1a) seems to have an advantage, when compared to the use of the CIS/Au-NP structure (Figure 1b), since Au nanoparticles can be embedded in the CIS without compromising the CIS phase. This conclusion may not strictly apply for a device, such as a solar cell, since the present study concentrated on the deposition and the optical properties of the materials only.

## Conclusions

Nanocomposite layers composed of CuInS_2_ (CIS) thin film and Au nanoparticles (Au-NP) were prepared by in-line chemical spray pyrolysis technique in two configurations: Au-NP/CIS and CIS/Au-NP both on a glass substrate. According to XRD, the spray of 2mM HAuCl_4_ aqueous solution with a volume of 2.5 to 15 ml onto a substrate at 340°C results in metallic Au nanoparticles. Irrespective of the volume sprayed, the Au crystallite size was in between 30 and 38 nm when Au particles are grown onto glass and in between 15 and 20 nm when grown onto CIS film. Agglomerates of Au nanoparticles with a size up to 200 nm are formed at spray volumes of 15 ml. According to XRD, the spray of HAuCl_4_ solution onto CIS causes damage of the CuInS_2_ film. We presume that the thermal decomposition products of HAuCl_4_ such as Cl_2_ and HCl react destructively with CuInS_2_.

The Au-NP/CIS nanocomposite films show additional light absorption in the absorbing region of the CuInS_2_ absorber material, characterized by a distinctive absorption band in the spectral region of 500 to 800 nm. Presumably, the band is due to surface plasmon resonance effect.

In the Au-NP/CIS configuration, the use of 15-ml 2 mM Au precursor solution to obtain Au nanoparticles by spray at 340°C leads to a gain of absorptance of up to 4.5 times when compared to the CuInS_2_ film reference. Thus, the preparation of CuInS_2_/Au-NP nanocomposite absorber by simple chemical spray method is feasible, and enhancement of light absorption in a solar cell with such thin absorber layer could be expected.

## References

[1] Gan Q, Bartoli FJ, Kafafi ZH (2013). Plasmonic–enhanced organic photovoltaics: breaking the 10% efficiency barrier. Adv Mater.

[2] Catchpole KR, Polman A (2008). Plasmonic solar cells. Opt Express.

[3] Ferry VE, Verschuuren MA, Li HBT, Verhagen E, Walters RJ, Schropp REI, Atwater HA, Polman A (2010). Light trapping in ultrathin plasmonic solar cells. Opt Express.

[4] Reineck P, Lee GP, Brick D, Karg M, Mulvaney P, Bach U (2012). A solid-state plasmonic solar cell via metal nanoparticle self-assembly. Adv Mater.

[5] Wang L-D, Zhang T, Xsong Y-J, Song Y-J, Li R-Z, Zhu S-Q (2014). Optical properties of Ag nanoparticle-polymer composite film based on two-dimensional Au nanoparticle array film. Nanoscale Res Lett.

[6] Atwater HA, Polman A (2010). Plasmonics for improved photovoltaic devices. Nature Mater.

[7] Brown MD, Suteewong T, Kumar RSS, D'Innocenzo V, Petrozza A, Lee MM, Wiesner UI, Snaith HJ (2011). Plasmonic dye-sensitized solar cells using core-shell metal-insulator nanoparticles. Nano Lett.

[8] Meen T-H, Tsai J-K, Chao S-M, Lin Y-C, Wu T-C, Chang T-Y, Ji L-W, Water W, Chen W-R, Tang I-T, Huang C-J (2013). Surface plasma resonant effect of gold nanoparticles on the photoelectrodes of dye-sensitized solar cells. Nanoscale Res Lett.

[9] Schaub A, Slepička P, Kašparkova I, Malinsky P, Mackova A, Švorčik V (2013). Gold nanolayer and nanocluster coating induced by heat treatment and evaporation technique. Nanoscale Res Lett.

[10] Narayanan R, El-Sayed MA (2005). Catalysis with transition metal nanoparticles in colloidal solution: nanoparticle shape dependence and stability. J Phys Chem B.

[11] Pedrueza E, Valdés JL, Chirvony V, Abargues R, Hernández-Saz J, Herrera M, Molina SI, Martínez-Pastor JP (2011). Novel method of preparation of gold-nanoparticle-doped TiO_2_ and SiO_2_ plasmonic thin films: optical characterization and comparison with Maxwell-Carnett modeling. Adv Funct Mater.

[12] Oja Acik I, Dolgov L, Krunks M, Mere A, Mikli V, Pikker S, Loot A, Sildos I (2014). Surface plasmon resonance caused by gold nanoparticles formed on sprayed TiO_2_ films. Thin Solid Films.

[13] El-Deab MS, Sotomura T, Ohsaka T (2006). Oxygen reduction at Au nanoparticles electrodeposited on different carbon substrates. Electrochim Acta.

[14] de la Garza M, Hernández T, Colás R, Gómez I (2010). Deposition gold nanoparticles on glass substrate by ultrasonic spray pyrolysis. Mater Sci Eng B.

[15] Montero MA, de Chialvo MRG, Chialvo AC (2009). Preparation of gold nanoparticles supported on glassy carbon by direct spray pyrolysis. J Mater Chem.

[16] Ramgir NS, Hwang YK, Jhung SH, Kim H-K, Hwang J-S, Mulla IS, Chang J-S (2006). CO sensor derived from mesostructured Au-doped SnO_2_ thin film. Appl Surf Sci.

[17] Tarwal NL, Devan RS, Ma YP, Patil RS, Karanjkar MM, Patil PS (2012). Spray deposited localized surface plasmonic Au-ZnO nanocomposites for solar cell application. Electrochim Acta.

[18] Goossens A, Hofhuis J (2008). Spray-deposited CuInS_2_ solar cells. Nanotechnology.

[19] Oja Acik I, Katerski A, Mere A, Aarik J, Aidla A, Dedova T, Krunks M (2009). Nanostructured solar cell by spray pyrolysis: effect of titania barrier layer on the cell performance. Thin Solid Films.

[20] Krunks M, Kärber E, Katerski A, Otto K, Acik IO, Dedova T, Mere A (2010). Extremely thin absorber layer solar cells on zinc oxide nanorods by chemical spray. Sol Energy Mater Sol Cells.

[21] Kärber E, Abass A, Khelifi S, Burgelman M, Katerski A, Krunks M (2013). Electrical characterization of all-layers-sprayed solar cell based on ZnO nanorods and extremely thin CIS absorber. Sol Energy Mater Sol Cells.

[22] Otto K, Oja Acik I, Krunks M, Tõnsuaadu K, Mere A (2014). Thermal decomposition of HauCl_4_⋅3H_2_O and AgNO_3_ as precursors for plasmonic metal nanoparticles. J Therm Anal.

[23] Wang W, Cassar K, Sheard SJ, Dobson PJ, Bishop P, Parkin IP, Hurst S, Bittnar Z, Bartos PJM, Němeček J, Smilauer V, Zeman J (2009). Spray deposition of Au/TiO2 composite thin films using preformed nanoparticles. Nanotechnology in Construction.

[24] International Centre for Diffraction Data (ICDD) (2008). Powder diffraction file (PDF), PDF-2.

[25] Repän T, Pikker S, Dolgov L, Loot A, Hiie J, Krunks M, Sildos I (2014). Increased efficiency inside the CdTe solar cell absorber caused by plasmonic metal nanoparticles. Energy Procedia.

